# Comparison of different tracheal intubation methods for unstable upper cervical spine injuries in a human cadaver model

**DOI:** 10.1038/s41598-025-09724-2

**Published:** 2025-07-07

**Authors:** Davut Deniz Uzun, Niko R. E. Schneider, Shiyao Liao, Felix C. F. Schmitt, Markus A. Weigand, Michael Kreinest, Erik Popp, Frank Weilbacher

**Affiliations:** 1 Medical Faculty Heidelberg, Department of Anesthesiology, Heidelberg University, Heidelberg, Germany; 2 Department of Anesthesiology, Giessen University Hospital, Giessen, Germany; 3 Department of Orthopedic Surgery, Zhejiang Provincial People’s Hospital and People’s Hospital of Hangzhou Medical College, Hangzhou, China; 4 BG Trauma Center Ludwigshafen, Ludwigshafen, Germany

**Keywords:** Tracheal intubation, Cervical spine, Flexible bronchoscopic intubation, Direct laryngoscopy, Video laryngoscopy, Experimental models of disease, Musculoskeletal system

## Abstract

In severe trauma, it is estimated that approximately 2% of patients will sustain a spinal cord injury. The optimal method for advanced airway management that will minimize any associated cervical spine movement remains a topic of debate. Therefore, the aim of this study is to compare the effects of different tracheal intubation techniques in unstable injuries of the cervical spine. Tracheal intubation using conventional laryngoscopy (CL), video laryngoscopy (VL) or flexible bronchoscopic intubation (FO) was performed in six fresh human cadavers. Compression of the dural sac as well as angulation, distraction and intubation time were assessed by myelography in the presence of isolated atlanto-occipital dislocation (AOD) and of combined atlanto-occipital dislocation with atlanto-axial instability (AAI). In case of an isolated AOD, FO intubation resulted in significantly less compression of the dural sac at both levels compared to CL (− 0.46 mm vs. − 1.31 mm; *p* < 0.001, r = .66) for C0/C1 and (− 0.09 mm vs. − 0.19 mm; *p* =  < 0.05, r = .36) for C1/C2 and VL (− 0.46 mm vs. − 0.64 mm; *p* =  < 0.05, r = .42 for C0/C1 and (− 0.09 mm vs. − 0.22 mm; *p* =  < 0.01, r = .52) for C1/C2. Atlanto-axial Angulation in simultaneous AOD and AAI, the differences between CL and VL were significantly in favor of VL (4.1° vs. 3.2°; *p* =  < 0.05, r = .39), and using FO resulted in less angulation than CL (2.5° vs. 4.1°; *p* =  < 0.001, r = .60) and VL (2.5° vs. 3.2°; *p* =  < 0.05). FO required longer in the case of combined AOD and AAI (FO 16.6 s vs. CL 9.8 s; *p* =  < 0.001, r = .56), (FO 16.6 s vs. VL 9.7 s; *p* =  < 0.001, r = .56). The study demonstrated that tracheal intubation using VL caused significant less compression of the dural sac than the CL. FO showed the lowest compression at all measuring points, but took almost twice as long. For elective or stable patients, where time to airway management is not a relevant factor, FO appears to be the safest method. However, FO is not available everywhere, and in urgent emergency situations, the longer duration may not be acceptable. In such cases, video laryngoscopy can represent a compromise between duration and patient safety, and most physicians have more clinical experience with VL than with FO.

**Supplementary Information:**

The online version contains supplementary material available at 10.1038/s41598-025-09724-2.

## Introduction

In Europe, over 30.000 individuals sustain a traumatic injury to their spine annually. In approximately 25% of these cases, an associated injury to the spinal cord results in a neurological deficit^1^. Injuries to the cervical spine are associated with a high degree of morbidity and mortality. With 15/100,000 cases per year, a fracture of the cervical spine is not a rare injury. 30% of injuries to the cervical spine affect the upper cervical spine between the occiput and the second cervical vertebral body (C2)^2,3^. In the context of multiple injuries, the involvement of the cervical spine must also be considered, as at least 6% of severely injured patients exhibit instability of the cervical spine and about 4% suffer from injury to the cervical spinal cord^4,5^. It is estimated that 3–25% of spinal cord injuries occur after the initial trauma event^6^. According to existing guidelines the initial prehospital management of trauma patients focuses on managing airway, breathing and circulation as well as immobilization of the upper cervical spine. Advanced airway management is unavoidable in many cases^7^. However, according to the literature, airway management methods themselves may lead to an aggravation of the injury and deterioration of the neurological status^8^. These spinal cord injuries can have significant physical, psychosocial and socioeconomic consequences for patients and society as a whole^9^.

Over the last decades, different tracheal intubation techniques have emerged^10^. Conventional laryngoscopy (CL), also known as direct laryngoscopy (DL) is still a tool that’s most available, although video laryngoscopy (VL) is becoming more and more available in the prehospital setting and could be of advantage^11,12^. The currently valid German guidelines for prehospital airway management even recommend the use of the VL on the first attempt for any prehospital tracheal intubation^13^. In the context of prehospital airway management for trauma patients, a number of factors have the potential to complicate the procedure. In addition to the possibility of an unstable cervical spine injury, contamination or flooding of the airway by tissue, vomit, secretions, blood, etc. can make management extremely difficult^14^.

Consequently, both national and European guidelines advocate the primary utilization of video-laryngoscopes for tracheal intubation in severely injured patients^15,16^. The most recent airway management guideline published by Gomez-Rios et al. and endorsed by the Spanish Society of Anaesthesiology and Resuscitation (SEDAR), recommends video-laryngoscopy-guided tracheal intubation with a hyperangulated blade and preconfigured stylet in such cases^16,17^. This method is not without its limitations. For instance, sunlight reflecting off the screen or airway contamination from blood or vomit has the potential to affect the view of the glottis^17,18^. In instances where an experienced physician is unavailable or laryngoscopy is not a viable option, the utilization of a supraglottic airway device (SGA) with reduced traction of the cervical spine is an alternative^16,19^.In addition, flexible bronchoscopic intubation (FO) can be used in patients with suspected or verified upper cervical spine injury. Only a few emergency medical services (EMS) provide the necessary equipment for FO, therefore it is used primarily in the clinical environment. The optimal tracheal intubation procedure for patients with unstable injuries of this nature is still unclear^9,20,21^. Retrospective studies failed to determine any new neurological deficiencies following intubation under CL and in-line stabilization^22^. It has been demonstrated that CL does result in a marginally elevated degree of cervical spinal movement during tracheal intubation when compared with VL. However, this does not appear to result in an increased risk of spinal cord compression^9^. It is recognized that VL can provide significant advantages, especially under difficult airway conditions or in the case of an in-line stabilized cervical spine^23,24^. However, the extant literature contains contradictory data. For example, in the context of intubation under general anesthesia with neuromuscular blockade and manual inline stabilization, the use of VL resulted in better visualization of the glottis but did not significantly reduce the movement of the non-pathological cervical spine in comparison to DL^25^. It is regrettable that recommendations from various professional associations for the management of the airway in patients with suspected or confirmed cervical spine injury will not be available until 2024^21^. Due to the acute care of trauma patients, a study to assess the influence of airway management in unstable cervical spine injuries is not only technically difficult to implement, but also methodically and ethically. Nevertheless, it is a clinically essential issue that, if poorly managed, can have devastating consequences for the patient^26^. One potential avenue for acquiring high-quality data on this subject is to undertake an evaluation of the topic using human unfixed cadavers. It should be noted, however, that these cadavers are not entirely comparable and are subject to certain limitations. The aim of the study was to investigate which advanced airway management method is best suited for patients with an unstable injury of the cervical spine using three different methods for tracheal intubation. The primary endpoint of the study was the change in dural sac width during tracheal intubation. Angulation, distraction and time until intubation were measured as secondary endpoints.

## Methods

The protocol for this study was approved and had provided permission by the ethics committee of the Rhineland-Palatinate Medical Association (Registration No. 837.156.16) and registered in the German Clinical Trials Register (DRKS 00010499). This prospective study was conducted in accordance with the relevant institutional guidelines and in compliance with the Declaration of Helsinki of 1975, as revised in 2013. Investigations were conducted on six human cadavers. The baseline characteristics of the cadavers are shown in Table 1. We recruited fresh cadavers from the body donation programme at the University of Heidelberg. Citizens voluntarily donated their bodies after death and gave written consent for the bodies to be used for medical research and teaching. The cadavers were frozen shortly after death and thawed to room temperature before conducting the trials to ensure that tissue properties were as realistic as possible. The cadavers medical history does not include any documented cases of head and neck pathologies, cancer, radiotherapy, or prior surgical interventions in cervical spine. Exclusion criteria were spinal diseases or trauma as well as previous cervical spine surgery in the medical history.

**Table 1:** The table shows the baseline characteristics of the related human cadavers.

Cadaver number	Sex	Age	Height (cm)	Body weight (kg)	BMI (kg/m^2^)
1	Male	89	183	90	26.9
2	Male	70	177	70	22.3
3	Female	60	169	58	20.3
4	Male	74	188	95	26.9
5	Female	89	172	65	22.0
6	Male	75	180	87	26.9

All airway interventions were conducted in an alternating sequence by two specialist anesthetists who possessed the additional qualification in emergency medicine. The cadavers were placed in random order by the Institute of Anatomy. The study team had no influence on this order, however, no study-related randomization took place. The following interventions were examined:

1. Tracheal intubation by conventional (direct) laryngoscopy (CL) using a Macintosh blade (size 3) and commercially available intubation stylet (Aerotube^®^—HUM, Lünen, Germany).
2. Tracheal intubation using a video laryngoscope (VL) (Ambu King Vision aBlade, non-channeled size 3 blade, Ambu GmbH, Bad Nauheim, Germany) with identically constructed intubation stylet.
3. Tracheal intubation using a portable, compact, flexible disposable endoscope system for flexible bronchoscopic (FO) airway management (aScope 3 Regular, Ambu GmbH, Bad Nauheim, Germany).

A Magill tracheal tube with an inside diameter of 7.5 mm was used for all tracheal intubations. The position of the tracheal tube was verified through fluoroscopy. The effectiveness of manual in-line stabilization (MILS) remains a subject of ongoing debate. Furthermore, the procedure varies around the world. For the aforementioned reasons and due to the lack of standardization, we decided not to perform MILS routinely as part of this study.

### Myelography and fluoroscopy

**Fig. 1:**
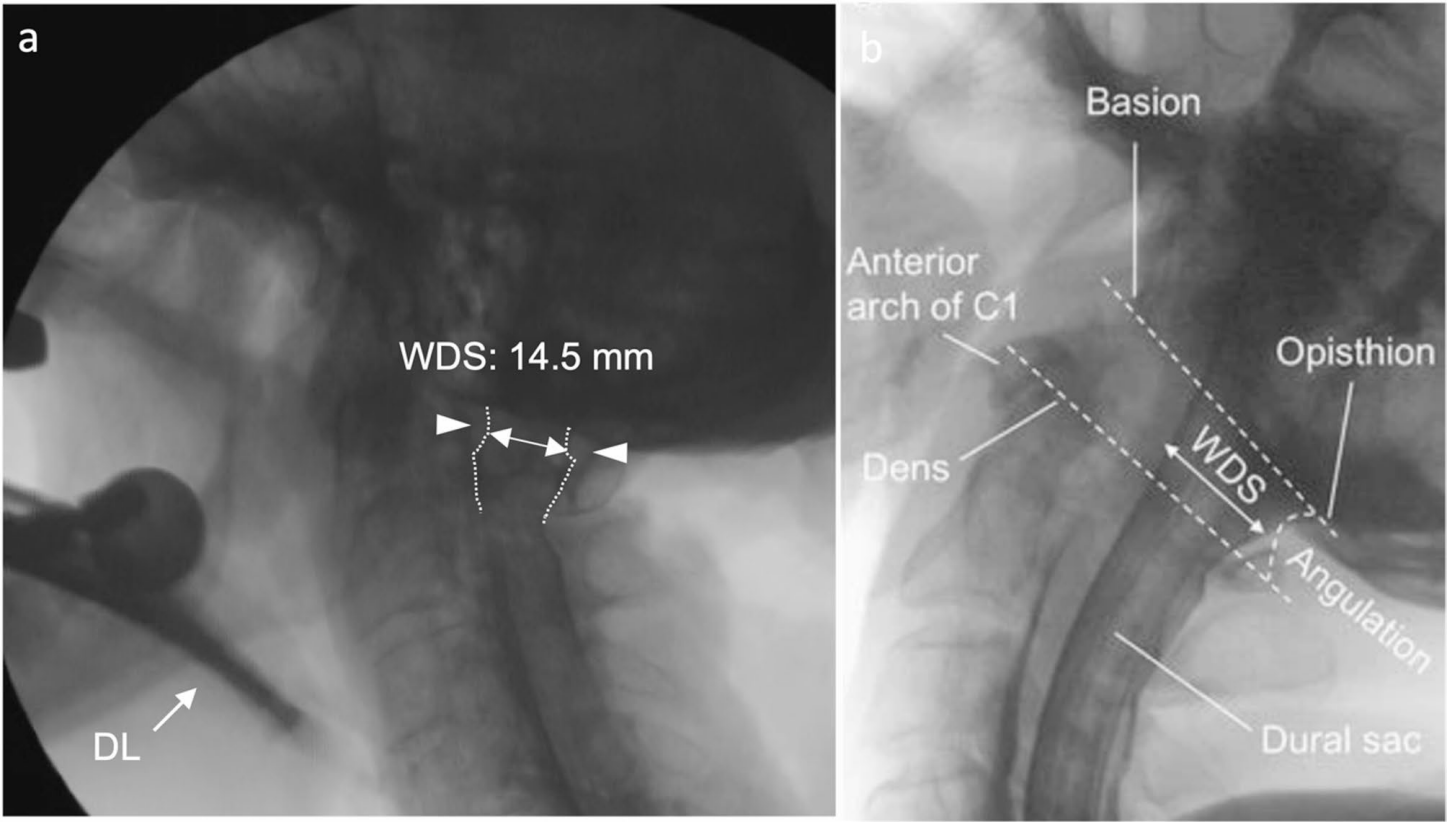
The figure (**a**) shows an example of a measurement of the myelography with the width of the dural sac (WDS) drawn in during tracheal intubation using direct laryngoscopy (DL). Section (**b**) showed the relevant anatomical landmarks for the measurements.

The bodies were placed in prone position prior to the interventions. The dural sac was exposed through a small incision at the level of the upper thoracic spine. Following this, a catheter was inserted intrathecally in cranial direction via a Tuohy needle. X-ray contrast agent (Optiray 300 mg/ml, Mallinckrodt, Germany) was administered through the inserted catheter using a syringe pump. The interventions to be examined were subsequently realized under lateral fluoroscopy using a C-arm (Veradius C-Arm, Philips, Netherland). Therefore, myelography provides unequivocal information regarding dural sac compression caused by soft tissues or bony structures^27^. Figure 1 shows an example of a measurement of the myelography.

### Measurements

**Fig. 2:**
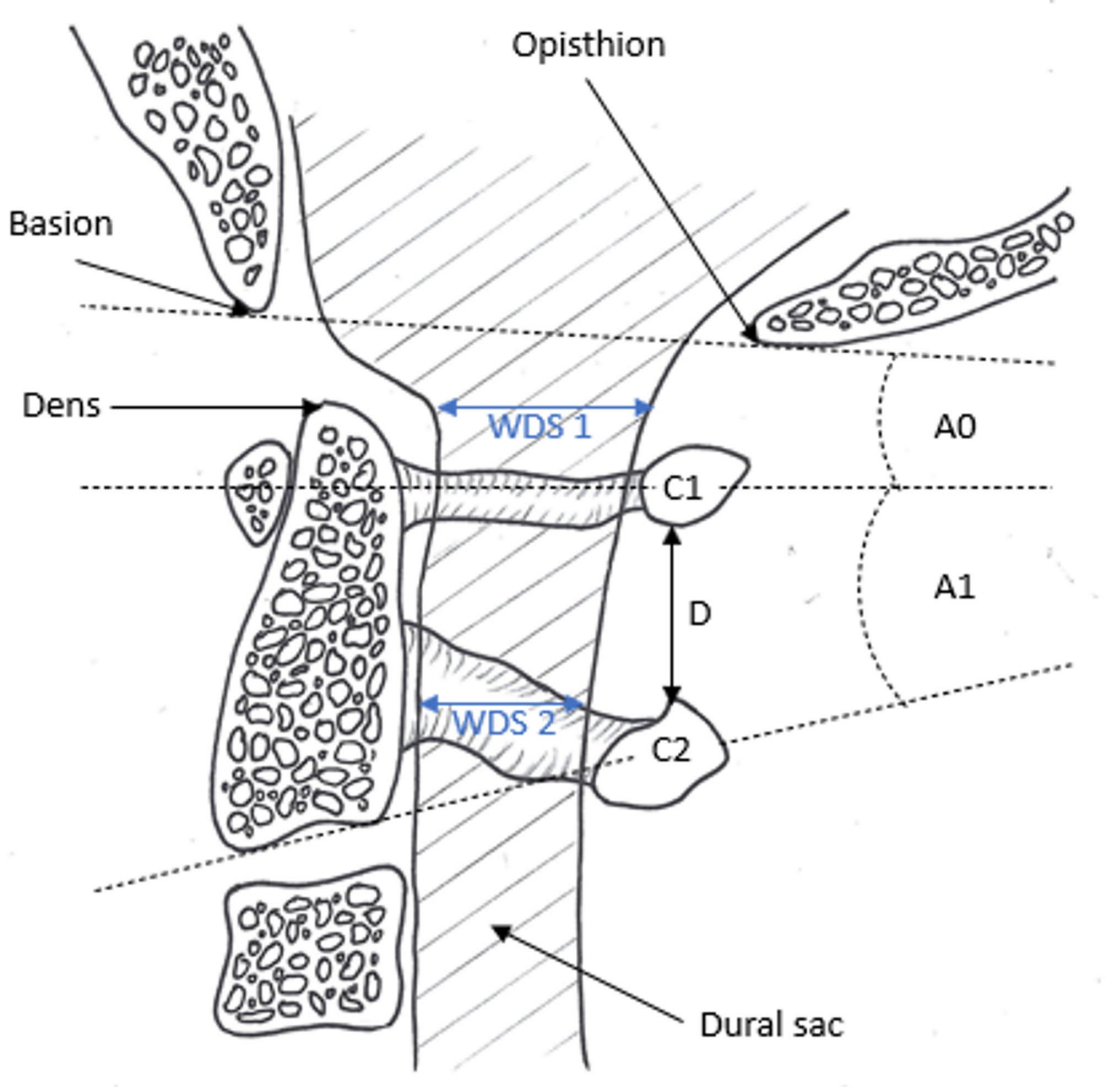
Anatomy of the upper cervical spine with important landmarks. The parameters measured in our study are marked (A0 and A1: Angulation; D: Distraction; WDS1 and WDS2: width of dural sac).

The primary endpoint of the study was the change in dural sac width during tracheal intubation. This was measured on the sagittal plane in the recorded fluoroscopy videos. The change in width of dural sac (WDS) was recorded in each case at the C1 and C2 level as the difference between the smallest width during the intervention and the width prior to commencing the intervention (WDS 1 and WDS 2; Fig. 2). Negative values in this case indicate compression of the dural sac.

Angulation, distraction and time until intubation were measured as secondary endpoints. Angulation was measured as the change in the angle of intersection of reference lines at each vertebra (Fig. 2). The angle of intersection of a line through the basion and opisthion (ventral and dorsal midpoint of the foramen magnum in the base of the skull) with the line viewed through the centers of the ventral and dorsal arc of C1 was used to determine angulation in the C0/C1 segment (A0; Fig. 2). In the C1/C2 segment, the angle of intersection of the line through the centers of the ventral and dorsal arc of C1 was adduced using the line through the base plate of C2 (A1; Fig. 2). The angles measured at the neutral cervical spine position served as initial values. Positive values indicate extension, with negative values indicating flexion. The perpendicular distance of the dorsal arc of C1 to the dorsal arc of C2 was measured to determine distraction in the C1 / C2 segment (D; Fig. 2). The respective initial value in the neutral position of the cervical spine was also determined here in each case. The intervention duration was determined by analyzing the fluoroscopy video recordings. No randomization or blinding was used. The first airway intervention was performed on cadavers with an intact cervical spine. Afterwards an atlanto-occipital dislocation (AOD) was induced surgically in all the cadavers through a dorsal approach as described before^28^.

Each airway technique used in each cadaver both before and after the injury was induced. Dislocation of the CO/C1 junction was confirmed by lateral video fluoroscopy during flexion and extension. The consensus statement of measurement for upper cervical spine injuries suggested that a basion-dental interval (BDI) of more than 12 mm was diagnosed as AOD^29^. BDI is measured through the distance between the basion and the tip of the dens. Dural sac was preserved intact in all cases, confirmed by myelographic measurements.

Following this, a ligamentary atlanto-axial instability (AAI) was additionally created on all the cadavers by expanding the operation previously performed. The atlanto-occipital joints were distracted again, therefore. The dural sac was then protected on the medial side and transverse ligaments were cut vertically at the level of the dens via both lateral atlanto-occipital cavities using a small scalpel. In addition, atlanto-axial joint capsules were opened and distracted using a small chisel. The instability of the injury was confirmed again through flexion and extension under lateral video fluoroscopy. The duration time was measured from the beginning of the tracheal intubation process until the tracheal tube was placed accordingly. The intra- and inter-observer reliability was ensured during the fluoroscopic measurements by the following points: (1) Exact definition of evaluation standards with uniform criteria for WDS, form, density or other characteristics. (2) Standardized image acquisition protocols, uniform scan parameters (e.g. same image resolution) and consistent patient positioning to reduce variations. (3) Staggered re-evaluation of own findings, the same observer re-evaluates the images at a later point in time to check consistency. (4) Education and training (Training on sample images with specified reference values).

### Statistical evaluation

The tracheal intubation methods were considered independently, and the differences examined pairwise according to Mann–Whitney. Missing data was not replaced, but rather considered as such. Absolute values were measured and used for evaluation. All three intubation procedures in each method and injury pattern were included in the analysis without any weighting. IBM SPSS Statistics 24 was used for statistical evaluation. We performed a preliminary study (data not shown), which showed a standard deviation of 0.22 mm for the change of the width of dural sac during airway management. We calculated a sample size of 18 interventions to detect a change in WDS of 0.2 mm with a power of 90% for α = 0.05. As this study was performed on a human cadaver model, no drop outs were anticipated.

## Results

### Changes in the width of dural sac

**Fig. 3:**
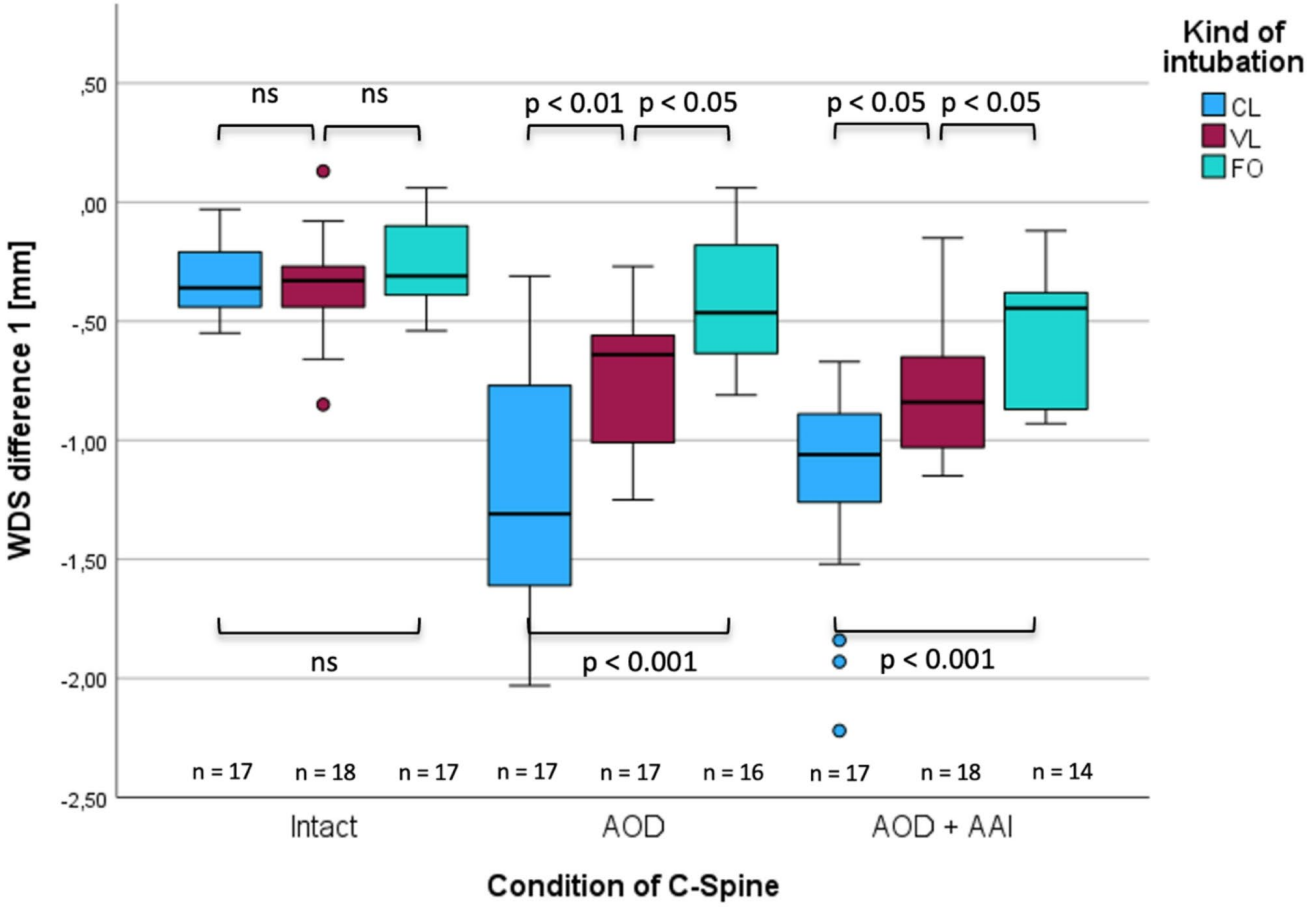
Changes in width of dural sac at the C0/C1-level (WDS1) in the intact and injured upper cervical spine during different tracheal intubation techniques.

**Fig. 4:**
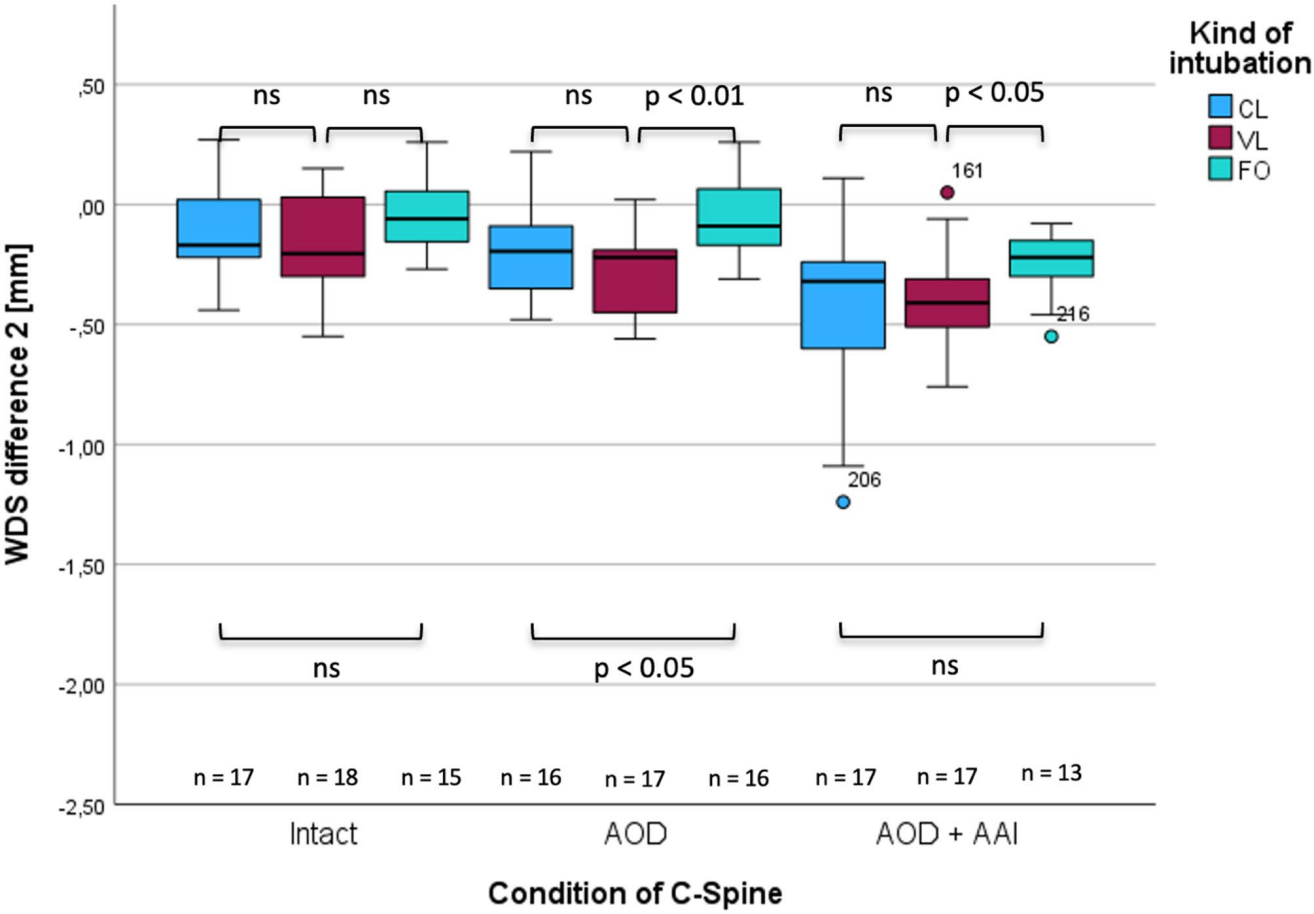
Changes in width of dural sac at the C1/C2-level (WDS2) in intact and injured upper cervical spine during different intubation techniques.

**Fig. 5:**
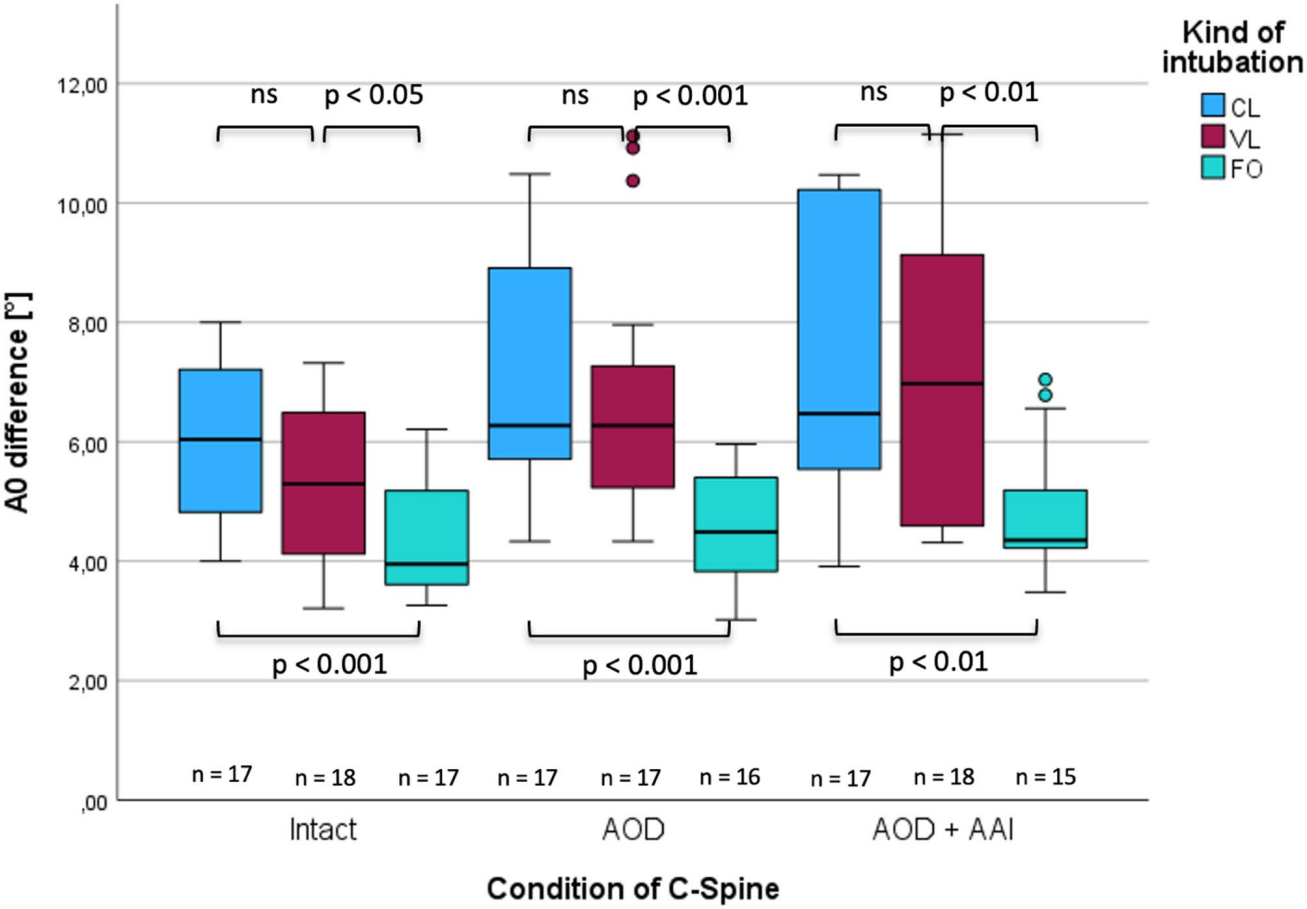
Changes in angulation at the C0/C1-level (A0) in intact and injured cervical spine during different intubation techniques.

In case of an intact spine, no significant differences were determined between the intubation techniques at C0/C1 level (Fig. 2; WDS1) CL − 0.36 mm, ± 0.15 mm vs. VL − 0.33 mm, ± 0.21 mm, (*p* = 0.961; ∆med = − 0.030), CL − 0.36 mm, ± 0.15 mm vs. FO 0.31 mm, ± 0.19 mm, (*p* = 0.339; ∆med = − 0.05) and at C1/C2 level (Fig. 2; WDS2) CL − 0.17 mm, ± 0.19 mm vs. VL − 0.20 mm, ± 0.20 mm, (*p* = 0.335; ∆med = 0.035); CL − 0.17 mm, ± 0.19 mm vs. FO − 0.60 mm, ± 0.15 mm; (*p* = 0.246; ∆med = 0.43; r = 0.20), respectively (Figs. 3, 4, and 5). The complete statistical analysis, including effect size (r), is provided in Table 2 in the supplementary material.

In case of an isolated atlanto-occipital dislocation, FO intubation resulted in significantly less compression of the dural sac at both levels compared to CL − 0.64 mm vs. − 1.31 mm, ( *p* = 0.009; ∆med = − 0.84, r = 0.66) for C0/C1 and − 0.09 mm vs. − 0.19 mm, (*p* = 0.039; ∆med = − 0.10, r = 0.36 for C1/C2, respectively, Figs. 3 and 4) and VL − 0.46 mm vs. − 0.64 mm, (*p* = 0.014; ∆med = − 0.17, r = 0.42) for C0/C1 and − 0.09 mm vs. − 0.22 mm (*p* = 0.002; ∆med = − 0.13, r = 0.52) for C1/C2, respectively).

VL resulted in significantly less compression than CL − 0.64 mm vs. − 1.31 mm; (*p* = 0.009, ∆med = − 0.67; r = 0.39) at C0/C1 level but failed to show a statistical significance at C1/C2 level − 0.22 mm vs. − 0.19 mm; (*p* = 0.276, ∆med = 0.02, r = 0.19). In case of simultaneous AOD and AAI, the use of FO for tracheal intubation resulted in significant less compression than with CL at the C0/C1 level − 0.44 mm vs. − 1.06 mm, (*p* =  < 0.001, ∆med = − 0.61; r = 0.71; Fig. 4) but failed to show a significance at C1/C2 level (*p* = 0.086, ∆med = − 0.22, r = 0.31; Fig. 5). The use of FO vs. VL resulted in significant less compression for both levels − 0.44 mm vs. − 0.84 mm, (*p* = 0.011; ∆med = − 0.39; r = 0.44) for C0/C1 and − 0.22 mm vs. − 0.41 mm (*p* = 0.035, ∆med = − 0.19; r = 0.38 respectively). VL resulted also in significant less compression than CL at the C0/C1 level − 0.84 mm vs. − 1.06 mm, (*p* = 0.019; ∆med = − 0.22, r = 0.39) but failed to do so at the C1/C2 level (*p* = 0.708, ∆med = − 0.41, r = 0.06).

### Atlanto-occipital Angulation (A0)

In the case of an intact upper cervical spine, no significant difference was evident between CL and VL (Fig. 5). FO intubation caused less angulation than CL (3.9° vs. 6.0°; *p* < 0.001, ∆med = 2.09, r = 0.60) and VL (3.9° vs. 5.2°; *p* = 0.035, ∆med = 1.34, r = 0.35). In the case of isolated atlanto-occipital dislocation, there was also no difference evident between CL and VL (∆med = 0, *p* = 0.865, r = 0.02), but using FO resulted in less angulation compared to CL (4.4° vs. 6.2°, *p* < 0.001, ∆med = − 1.78, r = 0.62) and VL (4.4° vs. 6.2°, *p* = 0.001, r = 0.57). In the case of simultaneous AOD and AAI, the differences between CL and VL were also insignificant (*p* = 0.568, ∆med = − 0.50, r = 0.10), and FO once again caused less angulation than CL (4.3° vs. 6.4°, *p* = 0.003, ∆med = 2.12, r = 0.50) and VL (4.3° vs. 6.9°; *p* = 0.002, ∆med = 2.62, r = 0.52; Fig. 5).

### Atlanto-axial Angulation (A1)

**Fig. 6:**
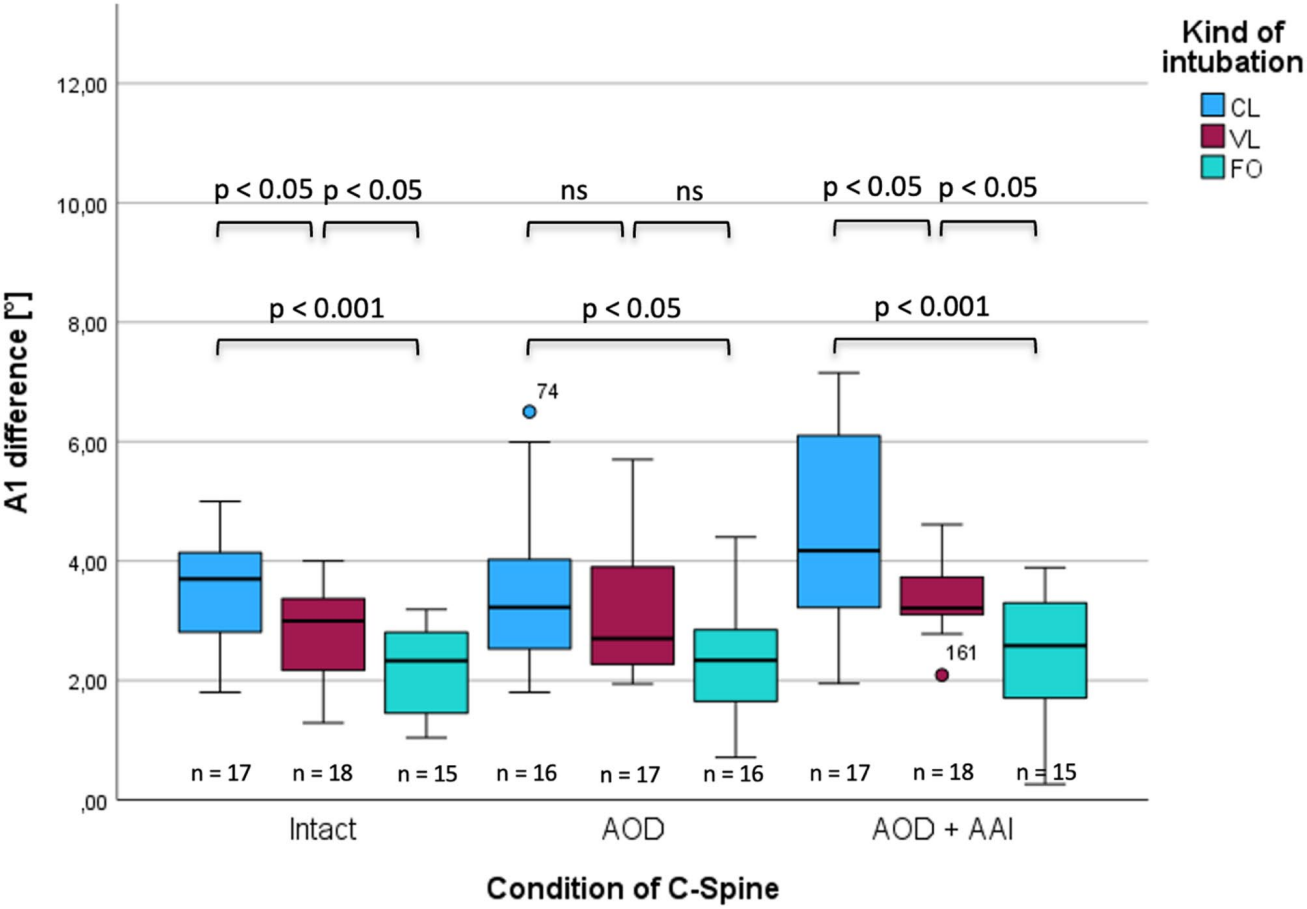
Changes in angulation at the C1/C2-level (A1) in intact and injured cervical spine during different intubation techniques.

In the case of an intact upper cervical spine, greater angulation A1 was evident during CL than during VL (3.7° vs. 2.9°, *p* = 0.038, ∆med = 0.70, r = 0.34; Fig. 6). Using FO resulted in less angulation than CL (2.3° vs. 3.7°, *p* = 0.001, ∆med = 1.37, r = 59) and VL (2.3° vs. 2.9°, *p* = 0.033, ∆med = 0.66, r = 0.37). In the case of isolated atlanto-occipital dislocation, there was also no difference evident between CL and VL (*p* = 0.423, ∆med = 0.52, r = 0.14). Again, using FO for intubation resulted in less angulation when compared to CL (2.3° vs. 3.2°, *p* = 0.026, ∆med = 0.89, r = 0.39) and VL (2.3° vs. 2.7°, *p* = 0.081, ∆med = 0.36, r = 0.29). In the case of simultaneous AOD and AAI, the differences between CL and VL were significantly in favor of VL (4.1° vs. 3.2°, *p* = 0.019, ∆med = 0.96, r = 0.39), and using FO resulted in less angulation than CL (2.5° vs. 4.1°, *p* = 0.001, ∆med = 1.59, r = 0.60) and VL (2.5° vs. 3.2°, *p* = 0.013, ∆med0.63, r = 0.42).

### Distraction

Regarding distraction, the measurements resulted in negative mean values for all the methods of intubation studied, which implies that the intubation resulted in an approximation and neck extension. No difference was evident on the intact spine between CL and VL (− 1.28 mm vs. − 1.12, *p* = 0.591; ∆med = − 0.16, r = ,10). Using flexible bronchoscopic intubation caused less extension than CL (− 0.80 mm vs. − 1.28 mm; *p* = 0.046; ∆med = − 0.48, r = 0.39) and VL (− 0.80 mm vs. − 1.12 mm, *p* = 0.032, ∆med = − 0.32, r = 0.41). In the case of isolated atlanto-occipital dislocation, no differences were evident between the different intubation procedures. Details are provided in the supplementary Table 2. In the case of a combination of AOD and AAI, no significant difference was evident between CL and VL (*p* = 0.310), and the FO resulted lower approximation than CL (− 0.97 mm vs. − 2.07 mm, *p* < 0.001, ∆med = − 1.10, r = 0.75) and VL (− 0.97 mm vs. − 1.76 mm, *p* = 0.002, ∆med = − 0.79, r = 0.57) with a high correlation.

### Tracheal intubation duration

CL and VL exhibited no statistical differences in intubation duration in either the intact or injured spine condition (10.5 s vs. 11.5 s *p* = 0.563, ∆med = − 0.98, r = 0.10). FO required longer in the case of an intact spine than CL (23.2 s vs. 10.5 s, *p* < 0.001; ∆med = − 12.74; r = 0.0.63) and VL (23.2 s vs. 15.5 s, *p* = 0.002, ∆med = − 11.76, r = 0.50). FO also required longer in the case of combined AOD and AAI (FO 16.6 s vs. CL 9.8 s, *p* = 0.001, ∆med = − 6.88, r = 0.57), (FO 16.6 s vs. VL 9.7 s; *p* =  < 0.001, ∆med = 6.9, r = 0.56). In the case of isolated atlanto-occipital dislocation, FO required more time than VL (17.4 s vs. 10.9 s; *p* = 0.050, ∆med = − 6.4, r = 0.33), with the significant difference between FO and CL (17.4 s vs. 11.1 s; *p* = 0.018, ∆med = − 6.21, r = 0.40).

The complete comparative analysis of all endpoints, including statistical analysis, is presented in Table S1 in the supplementary material.

## Discussion

This study describes movements of the injured upper cervical spine and compression of the structures inside the spinal canal in a human cadaver model. We employed myelography to display a possible compression of the dural sac at the level of injury. At this juncture, it is imperative to acknowledge that our study focused exclusively on upper cervical injuries, precluding us from offering any conclusions pertaining to lower spinal injuries. Furthermore, the potential for additional life-threatening complications arising from advanced airway management in cases of spinal cord pathologies underscores the paramount clinical significance of this subject^26^. It is also important to note that patients with a C-spine trauma may have other head and neck/facial traumas, which must be considered when choosing airway management strategies. There is also evidence in the literature that conventional laryngoscopy can increase the risk of injury to the cervical spine^15,30^. Therefore, the questions addressed in our study are of absolute clinical relevance for trauma patients.

Urgent tracheal intubation is a high-risk procedure with a high rate of adverse events^16,17,31^. Therefore, the anatomically difficult airway should be distinguished from the ‘physiologically difficult airway’. While the former is often considered in the context of airway management, the latter is often underestimated^32^. This is why national and international guidelines now address this concept^17^. Recognizing this concept can help to prepare for critical situations that may be avoidable or at least anticipatable with a little preparation. The focus here is on hypoxia, hypovolemia, acidosis, and right heart failure in critically ill or injured patients. Peri-intubation desaturation carries the highest risk of anesthesia-induced cardiac arrest. In this context, first-pass intubation success is of extreme clinical importance^33^. The incidence of peri-intubation complications increases fivefold after a second intubation attempt^34^.

We were able to demonstrate that dural sac compression and angulation at the C0/C1 level were unavoidable during airway management. In experienced providers those manipulations can be reduced to a minimum. Especially for prehospital EMS, where flexible bronchoscopic isn’t available, VL showed less compression of the dural sac when compared to CL at this level. Despite the fact that national and international guidelines recommend the primary use of the VL, it is not yet available in all EMS areas and countries^21^. Furthermore, the success rate of VL is contingent upon the device employed, the user’s experience and training with this airway aid. Consequently, in addition to the widespread implementation of the VL, regular training in controlled settings is imperative to ensure its reliability in high-risk areas such as prehospital emergency medicine.

In contrast to our study, a study by McCahon et al. was able to examine changes in vertebral canal dimensions during tracheal intubation in a cadaver model of atlantoaxial (type 2 odontoid process fracture) instability. The authors found no statistically significant differences in vertebral canal dimensions changes at the C1/C2 level between the Airtraq^®^ and Macintosh laryngoscopes or the Airtraq^®^ and McCoy laryngoscopes^35^. In a further comparative study, the performance of the Macintosh blade was evaluated in conjunction with three video laryngoscopes in two cadaveric models of severe cervical spinal instability^36^. In all levels ranging from C1 to C5, CL resulted in the most significant amount of vertebral body displacement. This was found to be considerably greater than the displacement produced by all three VLs. The vertebral levels (VLs) exhibited comparable degrees of vertebral body displacement. Consequently, these data, akin to our own, substantiate the primary utilization of VL in patients with suspected cervical spine injury. As demonstrated by Sawin et al. the greatest cervical movement during CL occurs in the atlanto-occipital and atlanto-axial area^37^. 30% of injuries occur between the occiput and C2 in the case of cervical spine fractures. Fractures of the first and second cervical vertebrae are most frequently encountered here, in contrast atlanto-occipital dislocation (AOD) is rare^2,3^. In the study, significantly less dural sac compression at the level of the first cervical vertebra was observed in both injury patterns under VL when compared to CL. No difference was evident in both methods at C2 level, so VL brought no further advantage here. No differences to CL were found in terms of angulation or distraction, which suggests that, even with VL, relevant movements can occur in the upper cervical spine. A review of the extant literature reveals considerable heterogeneity in the findings of previous studies in this area. The studies indicate a broad range of measurement methods, equipment used, immobilization techniques and injury patterns of the upper cervical spine^38–40^. Previous investigations also show a heterogeneous picture regarding the comparison of different systems for VL, so the transferability of the findings to other devices remains unclear^30,41^. The efficacy of airway management is contingent on numerous factors, including age, gender, the presence of concomitant diseases, and immobilisation of the cervical spine, amongst others^42–44^. In the present study, the cadaver was not subject to additional immobilisation; this factor may also influence the success of airway management and should be considered within the broader context.

Tracheal intubation using flexible endoscopes has been a standard method of advanced airway management in clinical anesthesia for many years^45^. A potential advantage of the flexible endoscopic method is the possibility to largely leave the cervical spine in the neutral position during the intubation procedure, leading to an appropriate intubation method for upper cervical spine injuries^46^. The availability of a flexible bronchoscope during intubation of severely injured patients is now demanded by national guidelines^47^. FO, which is only available in intensive care or emergency departments/trauma units, proved to be the best method towards compression of the dural sac and angulation but the duration of intubation doubled. This corresponds to the findings of other studies and must be considered when choosing the best method^48^. Furthermore, the selection of the optimal airway technique is contingent upon the patient’s clinical condition. To illustrate this point, consider a patient with a cervical spine injury who is hemodynamically stable and there is no acutely endangered airway. In such a case, the method that minimizes movement in the injured cervical spine is to be preferred^16,17^. In the context of semi-elective or elective airway management, awake flexible bronchoscopic tracheal intubation with extensive topical anesthesia is potentially the safest strategy. However, in emergency or urgent intubation scenarios, such as those involving a compromised airway or a hemodynamically unstable patient, the time component of the individual procedures must be taken into account. Based on our interpretation of the available data and our clinical experience, the approach recommended by the Spanish Society of Anaesthesiology and Resuscitation (SEDAR) for video laryngoscopy-guided tracheal intubation using a hyperangulated blade is the safest option in the majority of urgent cases. In the study, FO proved to be superior in isolated AOD as well as in combined AOD and AAI to both CL and VL in terms of dural sac compression. In addition, the FO caused the least angulation in the upper cervical spine in both injury patterns. These findings are consistent with studies conducted by other groups^45,49^. FO intubation in unconscious or anesthetized patients can be very effective in expert hands^16,50,51^. However, it is technically more demanding than in awake patients, is not fail-safe, and may result in episodes of desaturation or complete airway obstruction^52^. The presence of blood, vomit, or secretions in the airways of patients requiring emergency tracheal intubation further reduces the likelihood of success^16^. A current study of 1.177 patients with cervical spine injuries who underwent surgical fixation revealed a postoperative neurological complication rate of 0.34%^53^. The potential risk of a secondary spinal cord injury (for example, as a result of airway management) remains unknown. This may be attributable, at least in part, to delayed neurological deterioration, which occurs in approximately 10% of spinal cord injuries even when no clear causative factor can be identified^54^. This may result in a false association between advanced airway management interventions and subsequent neurological deterioration, despite the absence of direct evidence to support a causal relationship^9^.

Moreover, in the context of advanced airway management, additional measures, such as ventilation with a face mask, can result in further movement of the cervical spine^55^. The available historical evidence indicates that this phenomenon may be attributable to the movement of the head in order to facilitate the opening of the airway^56^. It is not possible to make comparisons in this context, as the present study has only examined the various tracheal intubation methods. Nevertheless, future studies should consider the entirety of the advanced airway management process in order to preclude the possibility of erroneous conclusions.

## Limitations

First, it was not possible to blind the provider nor the evaluator. As this study was conducted using X-ray the evaluator could see which airway device was used. Despite the fact that cadaveric biomechanical studies have reported no significant difference in cervical spine motion between intact fresh cadavers and living patients with both stable and unstable cervical spine conditions, it is important to note that cadaver studies are not without their limitations^57–60^. Des Furthermore, in cadaveric specimens, tissue fibrosis and edema, which can occur in trauma cases in living patients, must also be taken into account. The absence of soft-tissue responses, including reflex airway movements, is a limitation that restricts the generalizability of the findings to living patients. Second, many studies of airway management in cases of suspected cervical spine pathology make use of manual in-line stabilization (MILS) to restrict movement of the spine during airway maneuvers. However, the available data on the effectiveness of MILS in the stabilization of an injured cervical spine is limited^58,61^. Third, compression of the dural sac, angulation and distraction were used in this study as surrogate parameters for possible spinal cord damage. Clinical implications, such as neurological deficits, where changes in these parameters become relevant can only be tested on a living patient capable of neurological examination^62^. Since such investigations are prohibited for ethical reasons, the clinical relevance of the differences found cannot, ultimately, be conclusively assessed. Since even small changes in spinal canal width can presumably cause severe neurological damage, it is the authors recommendation to train and use the gentlest method available. The study examines tracheal intubation only, while preoxygenation, bag-mask ventilation, or patient positioning may also affect spinal stability. This should be considered with caution when interpreting the data. It is important to note that restrictions may also be imposed on techniques based on skill level and the geographical location or equipment available. However, in cases of trauma, this may be a pertinent consideration in determining the most suitable hospital for the patient. Another limitation is the small investigation with six cadavers and possibly the lack of randomization in the cadaver experiments. This lack of randomization could possibly influence the results through, for example, fatigue or tissue degradation.

## Conclusions

Advanced Airway Management in the case of patients with an unstable cervical spine requires a cautious approach to prevent secondary damage. Using an unfixed human cadaver model with isolated AOD and combined AOD/AAI, we managed to demonstrate that VL in both models causes significantly less compression of the dural sac than CL. Tracheal Intubation using FO caused the least dural sac compression in both models, both in comparison with conventional and video laryngoscopy. However, the intubation duration was at least doubled with FO. Flexible bronchoscopic intubation appears to be potentially the best intubation method in patients with an AOD or combined AOD/AAI. For elective or stable patients, where time to airway management is not a relevant factor, flexible bronchoscopic tracheal intubation appears to be the safest method for unstable upper cervical spine injuries. Unfortunately, this is not available everywhere, and in emergency situations, the longer duration may not be acceptable. In such cases, video laryngoscopy can represent a compromise between duration and patient safety, and most physicians have more clinical experience with VL than with FO. In light of the considerable heterogeneity among patients who require intervention following trauma, it is evident that a single airway management approach cannot be universally applied in all situations. It is probable that clinicians will be required to adapt their approach to airway management by selecting the most appropriate technique for each individual patient depending on his clinical and hemodynamic condition. It is also necessary to consider the treating physician’s experience of the individual advanced airway management method. In accordance with the prevailing data and in compliance with international guidelines, primary video laryngoscopy is recommended in the majority of urgent cases within the domain of emergency medicine.

## Electronic supplementary material

Below is the link to the electronic supplementary material.

Supplementary Material 1

## Data Availability

The datasets generated during and analyzed during the current study are available from the corresponding author on reasonable request.

## Abbreviations

A 0 and A 1: Angulation
AAI: Atlanto-axial instability
AOD: Atlanto-occipital dislocation
C1: First cervical vertebral body
C2: Second cervical vertebral body
CL: Conventional laryngoscopy
D: Distraction
DL: Direct laryngoscopy
EMS: Emergency medical services
FO: Flexible bronchoscopic intubation
MILS: Manual in-line stabilization
SGA: Supraglottic airway device
VL: Video laryngoscopy
WDS: Width of dural sac
